# Research-retreat-recovery: A potential model for organization and completion of research projects. Experience from a neurosurgery department in a developing country

**DOI:** 10.4103/2152-7806.72246

**Published:** 2010-10-30

**Authors:** Saniya Siraj Godil, Syed Faraz Kazim, Muhammad Shahzad Shamim

**Affiliations:** 1 Faculty of Health Sciences, Aga Khan University Medical College, Karachi, Pakistan; 2 Section of Neurosurgery, Department of Surgery, Aga Khan University Hospital, Karachi, Pakistan

**Keywords:** Developing country, neurosurgery, research

## Abstract

**Background:**

In the current era of biomedical research, it is imperative that every research study at an institution is properly organized, and frequently audited to streamline efforts and maintain standards. Recently, a research retreat was organized by the Section of Neurosurgery at Aga Khan University Hospital, Karachi, Pakistan, and following that a recovery team was made with the aim of recovering “lost in translation” research projects. In the realm of our experience, the current model is being proposed as a means for organization of departmental research.

**Methods:**

The “research” component of the model comprised compilation of an abstract book of all research work done within the section during the last five years. The “retreat” component of the model was intended with objectives of analysis of past research and generation of fresh ideas. The “recovery” component of the model was accomplished by formation of a research recovery team with the aim of recovering unfinished, and/or unpublished research projects.

**Results:**

The abstract book comprised 103 abstracts: 52.4% original research studies, 12.6% review articles, and 34.9% case report/series. Only 8.7% abstracts were of basic science research whereas the remaining 91.3% were clinical research papers. Only 34% had been published in an article form in a biomedical research journal (51.4% in international journals and 48.6% in national journals); remaining papers were either in submission/preparation process or had been abandoned. As part of research recovery, 29.4% projects were recovered within 12 weeks of the retreat component.

**Conclusion:**

We conclude that the model of “research-retreat-recovery” is highly successful in the context of neurosurgery departments in developing countries without a proper research unit, and can result in better organization of departmental research, recovery of unfinished projects, and initiation of new research studies.

## INTRODUCTION

Neurosurgery is acknowledged as a traditional craft; neurosurgeons, in general, were once considered to have little interest in academic research.[7] This was clearly highlighted in an article written by Richard Bergland in the New England Journal of Medicine, titled “Neurosurgery may die”, almost four decades ago.[1] Since then, the trends have changed, and neurosurgeons have made tremendous progress in the area of research, proving that neurosurgical research can not only help to devise the management guidelines of neurosurgical diseases but also lead to a better understanding of fundamental neurosciences and development of newer techniques.[10] Nowadays, special emphasis is laid on research in all neurosurgical training programs across the globe. This is evidenced by the increasing number of neurosurgical journals and gradual improvement in both the quality and quantity of neurosurgical publications.[2,3,5,6,8] On the other hand, research in Pakistan has shown little progress over the years.[2,9]

Research and publications have become the criteria for gauging the standard of an institution.[9] At a medical institution, everyone ranging from medical students to research officers, residents and faculty members is engaged in numerous research studies. There appears to be a global academic rush amongst the researchers to accomplish more and more in as little time as possible. It is therefore not unusual to find individuals working on several research projects simultaneously, leading to poor quality of research and publications. It is often seen that individuals within the same department work on overlapping projects resulting in a waste of time and resources. It is also not unusual to find a lot of research being lost prior to completion due to lack of interest, funding, or human resources.

**Figure 1:**
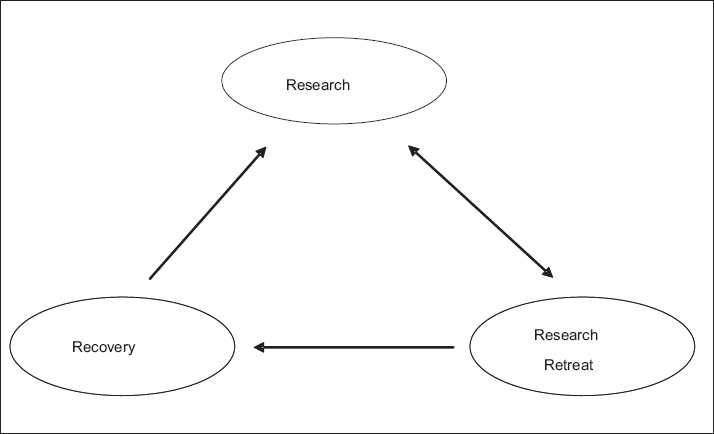
Model of research-retreat-recovery

It is, therefore, imperative that every research study at an institution is properly organized, and frequently audited to streamline efforts and maintain standards. In established academic institutions, this is done through a research coordination unit, research office, review boards, or any such research representative body with the objectives of proper organization of research activities. Establishment of such a unit facilitates further organization of research studies, but still does not ensure recovery of previous projects. Recently, a research retreat was organized by the Section of Neurosurgery at Aga Khan University Hospital (AKUH), Karachi, Pakistan, and following that, a research recovery team was made with the aim of recovering incomplete and lost research projects. In the realm of our experience, the current model is being proposed as a means for organization of departmental research in centers without a central research coordination unit or a prior research infrastructure [Figure 1].

## METHODS

### Setting

Aga Khan University Hospital is a tertiary care hospital in Karachi, the largest and most populous city of Pakistan. The University has a reputation for research interest. The Section of Neurosurgery, within the Department of Surgery, has been functioning as a postgraduate teaching unit since 2000, although until 2005 the program had only a single graduate to its credit. The section received a facelift in 2004, when it launched a formal structured neurosurgical postgraduate training program, with an inbuilt provision for research and capacity building. Over the last six years, the program has shown remarkable progress in research and has emerged as the leader of neurosurgical research within the country.

### Objectives and the model

The Neurosurgery Research Retreat 2010 (NR2010) was organized within the Section of Neurosurgery at AKUH, as part of the research-retreat-recovery model, in an attempt to:

1. Organize on-going research projects; analyze overall focus, strengths and weaknesses of projects.
2. Identify the different areas on which research has been conducted and others which have not yet been explored.
3. Generate fresh ideas and encourage new research projects by promoting multidisciplinary research collaboration.
4. Gain recognition of Section of Neurosurgery as a “Research Oriented Section”.
5. Recover unfinished, and/or unpublished research projects and submit them for publication.

The first two objectives were accomplished through the compilation and detailed analysis of all the research work done within the section over the study period, the “research” component of the model. The next two objectives were accomplished by the organization of NR 2010, and publication and distribution of the abstract book, the “retreat” component of the model. The final objective was accomplished by organizing a research recovery team, the “recovery” component of the model. The detail of each of these methods is hereunder.

### Research: data collection and abstract book

This component includes the research that is done within the section, as well as that which was done to compile, organize and analyze this information. For this purpose, all medical students, research officers, residents, faculty members and other individuals involved in any form of research with the section were contacted through e-mail and/or telephone. They were requested to submit abstracts of all research projects done during the last five years along with information regarding the current status of the project. Both international and local search engines such as PubMed/Medline and PakMediNet were also searched using key words “Neurosurgery” and “Aga Khan University” to identify published papers during the last five years. All locally published indexed and non-indexed journal issues were individually reviewed for neurosurgery-related publications from AKUH. Abstracts were also collected from the annual Health Sciences Research Assembly (HSRA) abstract books of AKUH. Compiled abstracts were then proof-read for accuracy and typographical errors, reformatted as per preset guidelines, and published in a hundred page NR2010 abstract book. To effectively organize the abstracts, the book was divided into seven sections: central nervous system (CNS) infections, spine, neuro-trauma, neuro-oncology, neuro-vascular, functional neurosurgery, and miscellaneous. The book also featured an author index and a list of neurosurgery-related oral presentations made by the section at various conferences during the last two years.

### Retreat: format of NR 2010

The retreat was organized within the University campus on a night just before a public holiday, and scheduled at a time when clinical services come to their conclusion; all in an effort to ensure maximum attendance. Invitees included the dean and associate dean of the university, chairs of various departments, faculty members and residents of sister specialties such as Neurology, Radiology, etc., neurosurgeons from other institutions as well as all researchers associated with the section. The theme of the event was “highlights of the epoch”, and it began with a brief overview of the section and its evolution into a research oriented unit, followed by oral presentations by neurosurgery residents on their best research project till date. This was followed by prize distribution for the best presentation and another for the best research idea based on the abstract book. The event lasted for 3 h and was concluded with an interactive poster exhibition and an informal dinner.

### Recovery: the team

Immediately following the retreat, a research recovery team comprising a neurosurgery faculty, a fresh medical graduate working as a research associate, and a medical student was organized. The recovery team had the objectives to:

1. Contact researchers with incomplete/unpublished research projects or those with unknown status of research projects.
2. Complete unfinished projects together with the principal researchers, or independently if the principal researchers consent to it.
3. Submit all completed projects for publication.

The team worked with deadlines and met on weekly basis. The outcomes reported in the present paper were achieved within 12 weeks of organizing the team.

## RESULTS

**Figure 2:**
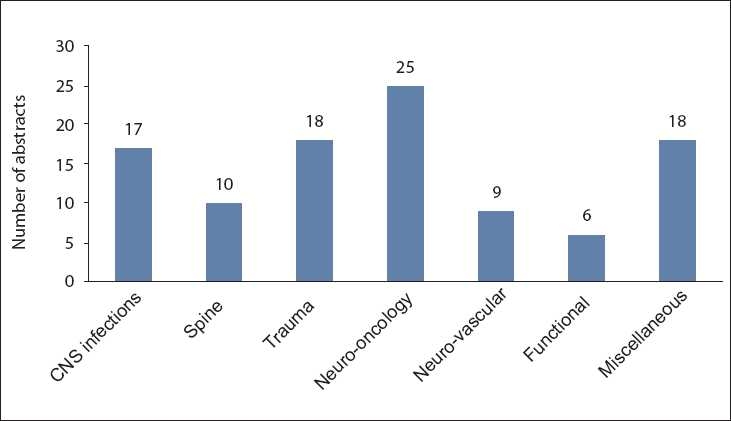
Different topics of research studies

A total of 103 abstracts of research projects involving 102 researchers were compiled. The researchers included faculty members, residents, research officers, and medical students. The abstracts included various aspects of neurosurgery: 17 (16.5%) focused on CNS infections, 10 (9.7%) on spine, 18 (17.5%) on neuro-trauma, 25 (24.3%) on neuro-oncology, 9 (8.7%) on neuro-vasular, 6 (5.8%) on functional neurosurgery and 18 (17.5%) on other miscellaneous topics [Figure 2].

**Figure 3:**
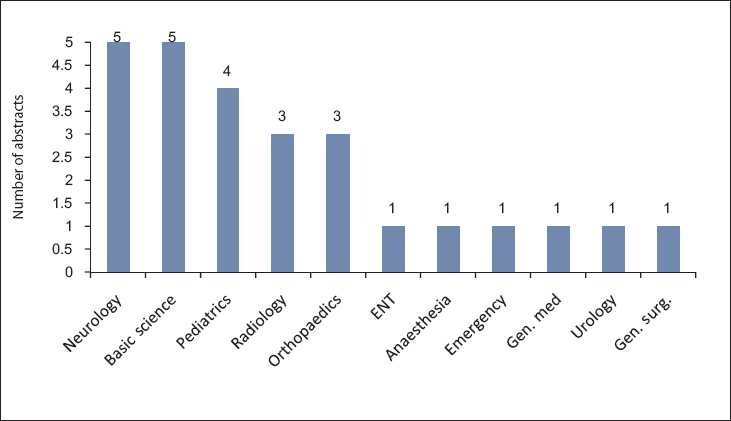
Different disciplines involved in research projects

Out of these 103 abstracts, 54 (52.4%) were original research studies, 13 (12.6%) were review articles, and 36 (34.9%) were case report/series. Only 9 (8.7%) abstracts were of basic science research whereas the remaining 94 (91.3%) were clinical research papers. A total of 26 (25.2%) research abstracts involved researchers from other disciplines including neurology (19.2%), basic sciences (19.2%), pediatrics (15.4%), orthopedics (11.5%), etc. [Figure 3].

Only 35 (34%) studies were published in biomedical research journals: 18(51.4%) in international journals and 17 (48.6%) in national journals. Of the 68 (66 %) unpublished studies, 21 (30.9%) were submitted for publication, 13 (19.1%) were complete but the manuscript was either unfinished or not submitted for various reasons, 24 (35.3%) were incomplete and 9 (13.2%) had been abandoned [Table 1]. Furthermore, 4 (3.9%) projects were found to be overlapping with other projects.

**Table 1:** Details of published and unpublished research projects

Status	Original research, Review article (*n*=67)	Case report/series (*n*=36)	Total number of papers (*n*=103)
Published	17 (25.4)	18 (50)	35 (34)
Unpublished	50 (74.6)	18 (50)	68 (66)
Submitted for publication	13 (26)	8 (44.4)	21 (30.9)
Complete, not submitted	10 (20)	3 (16.7)	13 (19.1)
Incomplete	19 (38)	5 (27.8)	24 (35.3)
Abandoned	7 (14)	2 (11.1)	9 (13.2)
Status unknown	1 (2)	0 (0)	1 (1.5)
Recovered	17 (34)	3 (16.7)	20 (29.4)
New Projects	11	3	14

Figures in parenthesis are in percentage

A total of 33 oral presentations in various research conferences were made by the neurosurgery section in the past two years with 18 (54.5%) presentations in year 2008 and 15 (45.5%) presentations in year 2009. Out of these 33 presentations, 6 (18.2%) were in international conferences while 27 (81.8%) were in national conferences across the country.

As part of research recovery, all the incomplete research projects were reviewed and 26 (25.2%) researchers were contacted and reminders were sent regarding completion of their projects. As a result of this, 8 projects were completed and another 10 which were already complete, were sent for publication with the help of the recovery team. The recovery team also helped to complete two original research projects which were previously abandoned. Thus, a total of 20 (29.4%) studies were recovered within 12 weeks of the organization of retreat. Furthermore, 11 new original research studies were initiated. This included a prospective spine surgery study and another one dealing with urological management of patients with spinal cord injury, designed with the help of urologists. Grants are now in process for both these projects. A new basic science project focusing on the role of ion channels as molecular target for gliomas was also initiated. A grant for establishment of a state-of-art brain tumor tissue bank was also updated and re-applied with the help of the recovery team.

## DISCUSSION

Neurosurgery is a rapidly progressing surgical specialty and research is an integral component of this process. In recent times, neurosurgery departments worldwide have tried to inculcate a culture of combining clinical excellence with excellence in research. The efforts for producing high quality research are streamlined by proper research units in Western countries. However, the scenario is quite different in developing countries like Pakistan, where the paucity of funding precludes the hiring of separate research faculty. There is a lack of research infrastructure in majority of medical specialities in Pakistan. This is even truer for sophisticated specialties such as neurosurgery. Most neurosurgery departments in the country lack proper research faculty which significantly affects the quality of research and the level of evidence produced by these units. In this context, our “research-retreat-recovery” model provides a cost effective solution to such shortcomings.

The research-retreat-recovery model has given us an opportunity to put into perspective our research efforts over the past five years and to analyze our strengths and weaknesses. The research retreat highlighted that most of the research studies focused on CNS infections, neuro-trauma and neuro-oncology while other areas were neglected. In this era of growing technology, newer techniques of minimally invasive neurosurgery are being introduced and there is a need of supportive evidence to prove the effectiveness of these approaches. The arena of research for neurosurgeons is pretty vast as many aspects of nervous system remain unexplored yet, and the intricacies of the network of the brain are poorly understood. The essence of research in neurosurgery is fundamental neurosciences but unfortunately, in our part of the world, there is paucity of basic science research and most of the research studies are clinically oriented.[2,9] This trend was obvious in our study. There were only a few prospective studies carried out in our department and case reports/series comprised a significant proportion of research and publications from our department. Similar trend of paucity of case-control studies, and high proportion of retrospective studies and case reports in neurosurgery worldwide has been mentioned in the literature as well.[4] There is definitely a need to conduct well-designed, prospective and case-control studies to contribute a higher level of evidence to neurosurgical literature. Many studies involved researchers from different disciplines including neurology, radiology, pediatrics, orthopedics, etc. This is a positive sign and its importance was emphasized in the retreat. Multidisciplinary approach and collaboration is essential for all health care institutions, not only for promotion of quality research but also for providing quality care to patients.

As part of the recovery component of our model, a research recovery team comprising a neurosurgery faculty, a research associate, and a medical student attempted to revive the projects which had been “lost in translation” due to one reason or the other. The retreat component had revealed that there were numerous research studies which were unpublished, incomplete or abandoned, and there were overlapping projects as well within the department. A strict need for channelizing research and completing unfinished projects was thus identified. The recovery rate of 29.4% within 12 weeks of the retreat is quite encouraging. The results seem even more pleasing when the lack of a proper research unit in our department is taken into account.

Due to the success of the research retreat, it has now been made an official, annual event of the Section of Neurosurgery at AKUH. Other surgical specialties have also been motivated to organize research activities within their own sections. Newer collaborations with the departments of neurology, neuro-radiology, anesthesia and basic sciences have been formed resulting in regular organization of a bimonthly Neuroscience Research Meeting, which is serving as a common forum to discuss neurosciences research projects prior to initiation, encouraging multidisciplinary input and collaboration. Special seminars and training courses for researchers will be planned in future to teach research methodology and statistical analysis to all those interested in research. An official departmental request for a full time statistician has also been forwarded by the Section of Neurosurgery.

We conclude that the model of “research-retreat-recovery is highly successful in the context of neurosurgery departments in developing countries and can result in better organization of departmental research, recovery of previous, unfinished projects, and initiation of new research studies.
